# Associations of Urinary Heavy Metal Mixtures with High Remnant Cholesterol among US Adults: Evidence from the National Health and Nutrition Examination Survey (1998–2018)

**DOI:** 10.3390/toxics12060430

**Published:** 2024-06-13

**Authors:** Hui Li, Bei-Jing Cheng, Pei-Yan Yang, Chun Wang, Ke Meng, Tian-Lin Li, Jia Wang, Ran Liu

**Affiliations:** Key Laboratory of Environmental Medicine Engineering, Ministry of Education, School of Public Health, Southeast University, Nanjing 210009, China; xiaohuixxi@163.com (H.L.); ahyichengbeijing@163.com (B.-J.C.); fydyangpy@126.com (P.-Y.Y.); luckywchun@163.com (C.W.); mk199606@163.com (K.M.); ltl201735222@163.com (T.-L.L.); xueerdawa_wj@163.com (J.W.)

**Keywords:** heavy metals, remnant cholesterol, NHANES, cardiovascular risk, Bayesian kernel machine regression

## Abstract

The main objective of our study is to explore the associations between combined exposure to urinary heavy metals and high remnant cholesterol (HRC), a known cardiovascular risk factor. Utilizing data from the National Health and Nutrition Examination Survey (NHANES) from 1999 to 2018, we conducted a cross-sectional analysis of 5690 participants, assessing urinary concentrations of ten heavy metals. Ten heavy metals in urine were measured by inductively coupled plasma mass spectrometry (ICP-MS). Fasting residual cholesterol ≥0.8 mmol/L was defined as HRC (using blood samples). Statistical analyses included weighted multivariable logistic regression, weighted quantile sum (WQS) regression, quantile g-computation (qgcomp), and Bayesian kernel machine regression (BKMR) to evaluate the associations of heavy metal exposure with HRC. Stratified analyses based on individual characteristics were also conducted. Multivariable logistic regression found that the four metals (*OR _Q_*_4 vs. *Q*1_: 1.33, 95% *CI*: 1.01–1.75 for barium (Ba); *OR _Q_*_4 vs. *Q*1_: 1.50, 95% *CI*: 1.16–1.94 for cadmium (Cd); *OR _Q_*_4 vs. *Q*1_: 1.52, 95% *CI*: 1.15–2.01 for mercury (Hg); *OR _Q_*_4 vs. *Q*1_: 1.35, 95% *CI*: 1.06–1.73 for lead (Pb)) were positively correlated with the elevated risk of HRC after adjusting for covariates. In addition, all three mixed models, including WQS (*OR*: 1.25; 95% *CI*: 1.07–1.46), qgcomp (*OR*: 1.17; 95% *CI*: 1.03–1.34), and BKMR, consistently showed a significant positive correlation between co-exposure to heavy metal mixtures and HRC, with Ba and Cd being the main contributors within the mixture. These associations were more pronounced in younger adults (20 to 59 years), males, and those with a higher body mass index status (≥25 kg/m^2^). Our findings reveal a significant relationship between exposure to the mixture of heavy metals and HRC among US adults, with Ba and Cd being the major contributors to the mixture’s overall effect. Public health efforts aimed at reducing heavy metal exposure can help prevent HRC and, in turn, cardiovascular disease.

## 1. Introduction

Remnant cholesterol (RC), also known as triglyceride-rich lipoprotein cholesterol, consists of the cholesterol carried in very-low-density lipoproteins (VLDLs), chylomicrons (CM) remnants, and intermediate-density lipoproteins (IDLs) [1]. The Copenhagen General Population Study reported that high remnant cholesterol (HRC) is present in 20% of 106,937 individuals [2]. Emerging evidence indicates that RC is a causal risk factor for atherosclerotic cardiovascular disease [3,4]. Similar to the pathogenic mechanisms of LDL, remnant lipoproteins penetrate artery wall sub-endothelial spaces and bind to proteoglycans, which leads to cholesterol accumulation, plaque formation, and inflammation, showing a pronounced atherogenic effect [5,6]. In addition, HRC is closely associated with obesity, non-alcoholic fatty liver disease, and metabolic syndrome and is a significant independent predictor of progression type 2 diabetes [7,8,9]. To date, there is no consensus on the clinical treatment of HRC; thus, prevention of HRC may be an effective strategy. 

The concept of RC has been proposed in recent years, and most research has concentrated on the relationship between RC and cardiovascular disease, with few studies examining the factors that influence RC. Thus, we reviewed risk factors associated with dyslipidemia, including genetics, lifestyle, and environmental factors [10,11]. In recent years, the widespread and persistent exposure to metals has also caused concerns. Growing evidence, including epidemiology and animal experiments, has shown a link between metals and abnormal lipid profiles. Exposure to toxic metals, such as cadmium (Cd), lead (Pb), mercury (Hg), and arsenic (As), was significantly associated with abnormal blood lipids [12,13,14,15]. However, the exact pathophysiological pathways underlying the adverse effects of toxic metals on blood lipid profile are unclear. The main pathogenesis may be related to hepatic toxicity through mechanisms such as inducing oxidative stress, disrupting cellular signaling, and stimulating inflammation [16,17]. Several studies have reported that metals like Cd, Pb, and barium (Ba) are linked to hepatic toxicity in animals [18,19,20].

To date, existing research has primarily focused on the relationship between single metals or mixtures of metals and traditional lipid indicators such as total cholesterol (TC), triglycerides (TG), high-density lipoprotein cholesterol (HDL-C), and LDL-C, but to our knowledge, there is no published evidence of a relationship between metals and HRC. Thus, we utilized the National Health and Nutrition Examination Survey (NHANES) database, due to its substantial sample size, to examine the relationships between combined exposure to urinary metals and HRC in this study. This study could offer new insights into the associations between metal exposure and HRC risk. Our study aims to (1) assess the associations between combined heavy metal exposure and HRC, (2) identify the most impactful metals within the mixture on HRC, (3) explore interactions within the mixture, and (4) pinpoint susceptible subgroups.

## 2. Materials and Methods

### 2.1. Study Population

This study utilized data from the 1999–2018 NHANES. A total of 101,146 subjects participated across ten consecutive NHANES survey cycles from 1999 to 2018. Participants under 20 years old, pregnant, or with cancer were excluded from this study. Additionally, 42,704 participants were excluded due to missing information on ten urinary metals or RC. Figure S1 provides a detailed flowchart of the participant selection process. The NHANES protocols received approval from the National Center for Health Statistics (NCHS) ethics review board, and all participants provided written informed consent.

### 2.2. Evaluation of High Remnant Cholesterol (HRC)

Participants enrolled in the NHANES program were asked to extract a blood sample, which was kept frozen at −20 °C and analyzed at the University of Minnesota in Minneapolis, Minnesota. TC was measured using enzymatic assay [21]. Serum TG and HDL-C were quantified using photometry. LDL-C levels were derived using the Friedewald equation based on direct measurements of TC, TG, and HDL-C. RC levels were calculated by subtracting HDL-C and LDL-C from TC. Based on the joint consensus statement from the European Atherosclerosis Society and European Federation of Clinical Chemistry and Laboratory Medicine, HRC was defined as fasting RC ≥ 0.8 mmol/l, and normal RC (NRC) was defined as fasting RC < 0.8 mmol/l [22].

### 2.3. Metal Measurements

We selected ten heavy metals for analysis based on their consistent measurement in the NHANES database from 1999 to 2018 and their well-documented potential health risks. Urine samples from NHANES were analyzed for barium (Ba), cadmium (Cd), cobalt (Co), cesium (Cs), mercury (Hg), molybdenum (Mo), lead (Pb), antimony (Sb), thallium (Tl), and tungsten (Tu) using inductively coupled plasma mass spectrometry (ICP-MS), as detailed in the NHANES Laboratory Procedure Manual. This method ensures the reliability of our data. For metals with concentrations below the detection limit, we employed a standard imputation method using the square root of two to maintain data integrity. Furthermore, the urinary creatinine concentrations were utilized to adjust urine metal levels, expressing them as “μg/g”.

### 2.4. Potential Covariates

Confounding factors considered in the analysis included age (20–59 and ≥60 years) [23], gender, race/ethnicity, body mass index (BMI), education levels, family income-to-poverty ratio (PIR), marital status, serum cotinine, drinking status, HEI-2015, physical activity, hypertension, and diabetes. Serum cotinine levels were used to determine smoking status, with >14 ng/mL indicating smokers and ≤14 ng/mL indicating non-smokers, as specified in [24]. Drinking status was classified as “never” for individuals who had less than 12 drinks in a lifetime, “former” for those who had over 12 drinks in 1 year and did not drink last year or did not drink last year but drank more than 12 drinks in a lifetime, and “now” for those had drank every day. Physical activity levels were quantified using average metabolic equivalent task scores (METs), with intensity categories established at MET thresholds of 4 for moderate and 8 for vigorous activities. The overall physical activity score was derived by totaling weekly MET minutes for both activity intensities. Levels of physical activity were defined as ‘optimal’ for ≥1500 MET-mins/week, ‘moderate’ for 600–1500 MET-mins/week, and ‘low’ for <600 MET-mins/week. The quality of diet was evaluated according to the 2015–2020 Dietary Guidelines for Americans. Scores are categorized as optimal (≥70), average (50–70), and inadequate (<50). Hypertension was identified through blood pressure readings (systolic > 140 mmHg or diastolic > 90 mmHg), self-reported diagnoses, or the use of antihypertensive medications. Diabetes status was classified as ‘yes’ or ‘no’, diagnostic criteria including fasting plasma glucose ≥ 126 mg/dL, use of insulin or oral hypoglycemic agents, hemoglobin A1C ≥ 6.5%, or a self-reported diagnosis.

### 2.5. Statistical Analyses

The analysis considered the complex sample design, employing mobile examination center sample weights for estimate calculations. Weights for the aggregated NHANES survey periods were determined in accordance with the NHANES recommendations. Demographic characteristics across two groups (HRC and NRC) were compared using survey-weighted χ2 test for categorical variables and weighted linear regression models for continuous variables. To obtain an approximately normal distribution, the metal concentration was log-transformed. The correlation of pairs of log-transformed metals was assessed using Pearson correlation coefficients.

#### 2.5.1. Weighted Multivariable Logistic Regression

Weighted multivariate logistic regression models were employed to evaluate associations between single metals and HRC. In regression models, creatinine-adjusted heavy metals were analyzed both as continuous (log-transformed) and as categorical variables (quartiles), with the lowest quartile serving as the reference group. Each quartile’s median concentration was considered a continuous variable to examine the trend of quartiles. Two models were utilized in the research, with Model 1 accounting for age and gender adjustments and Model 2 including additional adjustments for race/ethnicity, BMI, education level, PIR, marital status, serum cotinine, alcohol consumption, HEI-2015, physical activity, hypertension, and diabetes. Covariates with missing data were classified as ‘missing’ for the analysis.

#### 2.5.2. Weighted Quantile Sum (WQS) Regression

Given the limitations of logistic regression in addressing mixture exposure problems, we opted for the WQS regression to analyze the impact of the combined exposure to 10 urinary metals on HRC [25]. WQS regression is adept at assigning weights to individual exposure variables, which allows us to evaluate their contribution to the outcome and identify potential high-risk factors. The model necessitates two runs to account for both positive and negative correlations, a feature that is essential for our analysis. The dataset was partitioned into a 40% training set for weight estimation and a 60% validation set to assess the significance of the WQS index, with the final index derived from the average weights across 1000 bootstrap samples.

#### 2.5.3. Quantile g-Computation (qgcomp)

Recognizing that WQS regression assumes a uniform directionality between exposures and the outcome, we selected the qgcomp regression to overcome this limitation [26]. Using the qgcomp model, we can evaluate the joint impact on the outcome when all exposures increase by one quartile synchronously, irrespective of the exposure–outcome association’s direction. When different exposures exert inverse directions impact on the outcome, qgcomp will allocate each exposure a weight value in their direction, and the total of different direction weights equals 1, respectively.

#### 2.5.4. Bayesian Kernel Machine Regression (BKMR)

Considering the potential non-linear and interactive effects that the aforementioned methods may not fully capture, we employed the BKMR analysis to assess the joint impact of all metals on HRC [27]. Specifically, BKMR model utilized a Gaussian kernel function, as detailed in Bobb et al. [28], which has been demonstrated to be flexible and effective for estimating exposure–response functions that include both non-linear and non-additive effects. To ensure convergence, the model was executed up to 10,000 iterations using a Markov chain Monte Carlo algorithm. The posterior inclusion probability (PIP) was computed to estimate the contribution of the mixture component towards the outcome, employing a threshold value of 0.5. The results of BKMR analysis were as follows: (1) joint effects of the metal mixture on the HRC risk, (2) the relative importance of mixture component, (3) non-linear and/or non-additive associations of individual metals with the HRC risk, and (4) interactive effects among mixture components.

#### 2.5.5. Subgroup and Sensitivity Analysis

Stratified analyses were conducted by age, categorizing individuals into young and middle-aged (20 to 59 years) and elderly (≥60 years), as well as by gender (male, female) and BMI (<25.0, ≥25.0 kg/m^2^). Furthermore, two sensitivity analyses were conducted to evaluate the consistency of the findings. Firstly, we applied weighted logistic regression models that incorporated each urinary metal to control for the influences of other metals. Secondly, false discovery rate (FDR) corrections were used to adjust *p*-values in weighted logistic regression to reduce the scenario of chance finding. Finally, multiple imputation was employed for covariates with missing values to assess baseline characteristics and the strength of our results. R (version 4.1.2; R Foundation for Statistical Computing) was utilized for statistical analysis. Two-sided *p* values below 0.05 were considered statistically significant.

## 3. Results

### 3.1. Baseline Characteristics

Of the 5690 participants in this study, 1408 (24.74%) were diagnosed with HRC. A total of 52.11% of the total population was female, and 70.23% of the participants were under the age of 60 (Table 1). In addition, significant differences in age, gender, race, BMI, education level, marital status, drinking status, hypertension, and diabetes were noted between the HRC group and non-HRC group participants, as shown in Table 1. Table S1 shows the urinary metal concentration distributions, detailing the detection rates, medians, and interquartile ranges. Detection rates of metals ranged from 74.01% to 99.96%. Pearson correlation coefficients for Ln-transformed metals showed moderate associations between Cs and Tl (r = 0.59) and Cd and Pb (r = 0.41), while other pairs exhibited weaker correlations (Figure S2).

### 3.2. Associations between Single Metal Concentrations and HRC: Logistic Regression

Table 2 shows the relationship between creatinine-adjusted metal concentrations and HRC, as determined by survey-weighted logistic regression models. After adjusting age and gender, we found that Ln-transformed Ba, Cd, and Pb (as continuous variables) had positive associations with HRC. After adjusting for all covariates, we observed similar results. In addition, Hg (*OR* = 1.09, 95% *CI*: 1.03–1.16) levels remained positively associated with HRC. Considering the possible non-linear correlation between individual metals and HRC, we categorized the metal concentrations into quartiles, using the lowest quartile as a reference, as shown in Table 2. After adjusting for age and gender, we only found that Ba, Cd, and Pb were significantly associated with 29% (95% *CI*: 1.01–1.65), 54% (95% *CI*: 1.23–1.93), and 28% (95% *CI*: 1.03–1.59) increases in HRC, respectively. After adjusting for all covariates, we found that Ba, Cd, Hg, and Pb were significantly associated with 33% (95% *CI*: 1.01–1.75), 50% (95% *CI*: 1.16–1.94), 52% (95% *CI*: 1.15–2.01), and 35% (95% *CI*: 1.06–1.73) increases in HRC, respectively. The results of the P-trend further support the above associations.

### 3.3. Associations between Co-Exposure to Metals and HRC: WQS, qgcomp, and BKMR Models

**Figure 1:**
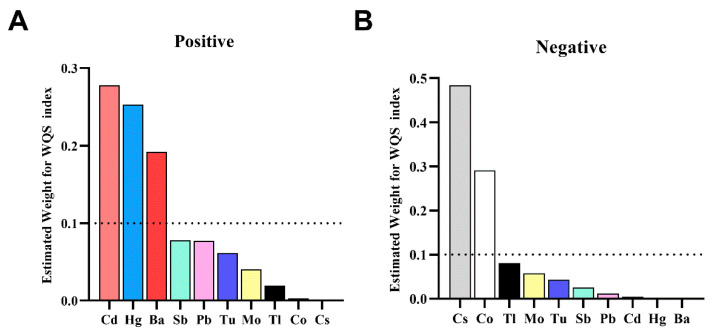
The weights of each metal in the WQS model for HRC. (**A**) for the directional of positive WQS model and (**B**) for the directional of negative WQS model. Adjusted variables included age, gender, race/ethnicity, body mass index, education level, PIR, marital status, serum cotinine, drinking status, HEI-2015, physical activity, hypertension, and diabetes.

The WQS regression displayed a positive correlation between the WQS index and the risk of HRC (*OR*: 1.25; 95% *CI*: 1.07, 1.46). Weighted values exceeding 0.1 were observed for Cd (0.278), Hg (0.253), and Ba (0.192) among urinary metals. When examining the negative directional association between urinary metal mixture and HRC risk, Cs (0.484) and Co (0.291) showed weighted values exceeding 0.1, as shown in Figure 1.

**Figure 2:**
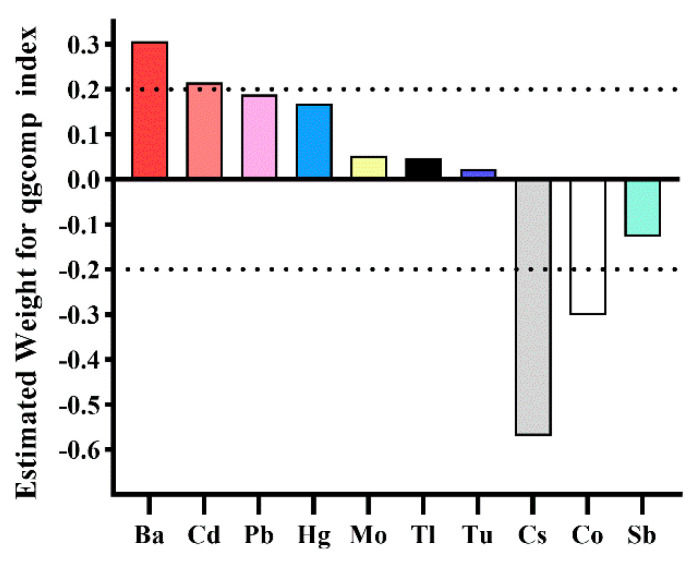
The weights of each metal in the qgcomp model for HRC. Adjusted variables included age, gender, race/ethnicity, body mass index, education level, PIR, marital status, serum cotinine, drinking status, HEI-2015, physical activity, hypertension, and diabetes.

Results from qgcomp also indicated a significant correlation between the urinary metal mixture and the HRC risk. With a one-quartile increase in the urinary metal mixture, the odds of HRC increased by 18% (*OR*: 1.18; 95% *CI*: 1.03, 1.34). Based on the weights of individual metals, Ba (0.307) and Cd (0.215) exceeded the threshold of 0.2 in contributing to the positive mixture effect, while Cs (0.570) and Co (0.302) surpassed this threshold in contributing to the negative overall effect, as detailed in Figure 2.

**Figure 3:**
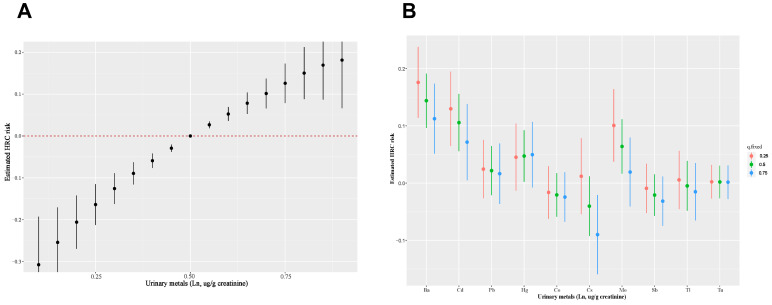
(**A**) Combined effects of urinary metal mixture on HRC risk were estimated by BKMR models. (**B**) Single metal exposure effect (95% CI) to HRC when other metals were fixed at a specific quantile (25th, 50th, 75th). Adjusted variables included age, gender, race/ethnicity, body mass index, education level, PIR, marital status, serum cotinine, drinking status, HEI-2015, physical activity, hypertension, and diabetes.

The BKMR model revealed a significant combined effect of the ten metals when all metals reached or exceeded the 55th percentile in comparison to their median values (Figure 3A). In the BKMR model’s metal mixtures, components with PIPs up to 0.5 included Ba (1.000), Cd (0.869), Mo (0.792), and Cs (0.553), as listed in Table 3. Moreover, our analysis indicated that when the other metals were fixed at different percentiles (25th, 50th, or 75th), a positive association between Ba, Cd, Mo, and HRC, while a negative correlation between Cs and HRC (Figure 3B). Furthermore, we further investigated the single metal dose–response curves and interaction among ten metals. In the univariate exposure–response analysis, Ba, Cd, Pb, and Hg displayed linear dose–response curves, whereas Cs and Mo showed U-shaped dose–response curves with other metals’ exposures fixed at the median, as depicted in Figure S3A. Bivariate exposure–response analysis suggested the absence of underlying interactions, as shown in Figure S3B.

According to the results of four statistical models, Ba and Cd emerged as the key metal risk factors for HRC among all participants, while Cs acted as a protective factor, as detailed in Table 3.

### 3.4. Subgroup and Sensitivity Analysis

After adjusting for all covariates, the WQS model revealed that joint associations were more pronounced among young and middle-aged participants (*OR*: 1.19, 95% *CI*: 1.00–1.40) and males (*OR*: 1.27, 95% CI: 1.04–1.54), as shown in Figure S4A. Consistent results were also found in qgcomp analysis, and Figure S4B showed that the combined effect was more significant in young and middle-aged adults (*OR*: 1.20, 95% *CI*: 1.02–1.41) and males (*OR*: 1.26, 95% *CI*: 1.05–1.51). In addition, subgroup analysis using the BKMR model further supported the above results, as presented in Figure S5. Interestingly, there was a positive trend in the groups with higher BMIs (BMI > 25 kg/m^2^) using BKMR methods, despite the fact that results of the WQS and qgcomp analyses were not significant (*OR*: 1.12, 95% *CI*: 0.96–1.31; *OR*: 1.12, 95% *CI*: 0.97–1.30), and the results were similar between them. Therefore, we also believe that metal mixtures have an impact on individuals with higher BMI.

Furthermore, after controlling for all covariates, three sensitivity analyses did not materially change our findings (Tables S2–S4; Figures S6 and S7).

## 4. Discussion

### 4.1. Key Findings

After controlling for all covariates, logistic regression displayed that Ba, Cd, Hg, and Pb (as continuous or categorized) were significantly positively associated with HRC levels. Analyses using WQS, qgcomp, and BKMR all revealed that exposure to a combination of heavy metals, specifically Ba and Cd, is significantly associated with elevated levels of HRC, while Cs of the mixture component was correlated with lower HRC levels. These associations were more pronounced in younger participants (20 to 59 years), males, and individuals with higher BMI (BMI ≥ 25 kg/m^2^). To our knowledge, our research marked the first investigation into the joint impact of metal mixtures on HRC. This study emphasized the critical importance of addressing environmental exposures in the prevention and management of cardiovascular risk factors and, in particular, the need to develop strategies to reduce exposure to heavy metals in the population.

### 4.2. Single Metals

In our research, we found the average urinary concentrations of Ba, Cd, and Cs to be 1.96 µg/L, 0.39 µg/L, and 5.46 µg/L (1.85 µg/g, 0.36 µg/g, and 5.02 µg/g), respectively. Ba concentrations were found to exceed the geometric mean of 1.10 µg/L and 1.48 µg/g observed in the general populations of China and the United States [29,30]. Cd levels were above the geometric means of the general populations in China, Germany, and the United States by 0.28 µg/L, 0.22 µg/L, and 0.21 µg/L, respectively [30,31,32]. Cs levels exceeded the general population geometric averages of 3.65 µg/L and 4.64 µg/g in China and the United States [29,30].

Limited studies were conducted to explore the link between urinary heavy metals and RC. Since RC was a type of cholesterol, we reviewed the literature that explored the association between metals and conventional lipid markers. Analogous to the results of most previous studies [33,34], our study also showed that Ba was positively associated with HRC. A large cross-sectional study based on NHANES identified urinary Ba as a risk factor for hyperlipidemia, in which the OR of the fourth quantile is 1.48 (95% CI: 1.17–1.87) compared with the lowest quartile [33]. In addition, a recent Chinese case–control study also showed a link between serum Ba levels and elevated TG and reduced HDL-C [34]. Furthermore, several studies have demonstrated a link between Ba exposure and metabolic disorders, including obesity and metabolic fatty liver disease [35,36,37]. The exact mechanism of the risk of HRC due to Ba is not known. Several studies have shown that exposure to Ba was positively correlated with liver injury, which is the primary site of cholesterol metabolism [19,38,39]. A study involving 3589 adults from America found a significant link between urinary Ba exposure and liver injury markers (alanine aminotransferase, aspartate aminotransferase, etc.) [38]. Oxidative stress and inflammation could be key factors in liver damage pathogenesis. Elwej et al. found that rats exposed to Ba exhibited oxidative stress in the livers, characterized by elevated levels of malondialdehyde, lipid hydroperoxides, and advanced oxidation protein products. This suggests the production of excessive free radicals following oxidative stress injury and an imbalance in the antioxidant defense system [19]. Based on previous research on how heavy metals affect the risk of hyperlipidemia, we proposed that Ba may increase this risk by initially disrupting the liver’s antioxidant system, subsequently impairing normal liver lipid metabolism functions. However, our hypothesis is not yet supported by sufficient evidence, highlighting the need for further investigation into these potential mechanisms.

Growing evidence has shown that exposure to Cd is an important environmental risk factor for lipid metabolism disorders [33,40]. We also found a significant link between exposure to Cd and HRC. A recent meta-analysis demonstrated that elevated Cd was correlated with increased TC levels (OR = 1.48, 95% CI: 1.10–2.01), increased LDL-C levels (OR = 1.31, 95% CI 0.99–1.73), and decreased HDL-C levels (OR = 1.96, 95% CI: 1.09–3.55) [41]. Several large-scale research studies have concluded that high Cd exposure increases the risk of dyslipidemia [33,42]. Most studies on the mechanism of Cd altering lipid metabolism have consistently shown that Cd interferes with the body’s antioxidant system. A study exploring the mechanism behind cadmium-induced liver toxicity in pigs revealed significant increases in both apoptosis of liver tissue cells and reactive oxygen species (ROS) levels following cadmium exposure [43]. Another possible explanation involved dysautophagy induced by Cd exposure, worsening hyperlipidemia in hepatocytes overloaded with cholesterol, which hinders triglyceride clearance [44]. Regardless, oxidative stress is pivotal in the potential toxicity triggered by Cd.

This study also revealed that elevated Cs level has a negative impact on HRC. However, limited research has investigated the effects of Cs exposure on human lipid profiles, and most have concentrated on the association between exposure to Cs and metabolic syndrome and its diagnostic factors. Two US population-based studies consistently indicated that Cs exposure was negatively related to metabolic syndrome [45,46]. When Zha et al. further analyzed the effect of Cs exposure on its components, Cs had the largest weight in TG in the negative direction of the WQS model [46]. We hold that since RC is TG-rich lipoprotein cholesterol, a decrease in TG also leads to a decrease in RC. Studies on the mechanism of Cs exposure are limited. An animal study has revealed a decrease in apolipoprotein E in rat liver after nine months of Cs exposure [47]. Apolipoprotein E mainly exists in CM, VLDL, IDL, and some HDL, and lowering Apo E concentration results in decreased plasma triglyceride levels. This can also indirectly reflect the negative relationship between Cs and RC. The current lacking related studies impedes researchers’ ability to compare findings. Consequently, further study is needed to explore and clarify the link between Cs exposure and lipid metabolism.

### 4.3. Combined Exposure

Given the complex synergistic or antagonistic interactions among metals, traditional statistical methods may not be able to show the true relationship between metal mixtures and HRC. Consequently, we applied three primary statistical methods for mixture analysis, including WQS, qgcomp, and BKMR, to investigate associations of co-exposure to metal mixtures with HRC. As expected, all three methods consistently showed that metal mixture is positively correlated with HRC risk. Analogous to our findings, several researchers, such as Zhao et al. [12] and Wang et al. [33], have shown that exposure to a mixture of heavy metals is correlated with markers of dyslipidemia. Although metal mixtures have been known to have an influence on HRC, their relative importance is unknown. After a comprehensive comparison of the results of the four methods, we identified that Ba and Cd are the main positive contributors toward HRC risk, while Cs is the main negative contributor.

### 4.4. Subgroup Analysis

In subgroup analysis, we noted a more obvious effect of metal mixture exposure in young and middle-aged adults (20~59 years), which was similar to several previous research studies [23,33,48]. One possible explanation is that the increase in baseline oxidative stress levels in older adults may have masked the effect of elevated levels of heavy metal-induced oxidative stress, thus confusing the association between toxic metals and hyperlipidemia and leading to differences in subgroup analysis results. Moreover, we also observed that males were more affected by the metal mixture. This might be partly attributable to the fact that males are more likely to work occupations that involve heavy metal exposure compared with females. Moreover, males also tend to have higher exposure to smoking, which is generally recognized as an important source of heavy metal exposure. In our study, our results supported the above hypothesis, in which males have higher serum cotinine concentrations (mean: 77.64 vs. 44.82 ng/mL). In line with our expectations, individuals with higher BMI status (BMI ≥ 25 kg/m^2^) were more susceptible to metal mixtures. Heavy metals can accumulate in fatty tissue, which prolongs the presence of heavy metals in the body. Therefore, adults with higher BIM status suffer longer effects of heavy metals. However, more research is needed to verify these hypotheses in the future.

### 4.5. Implications for Public Health

Our study’s findings emphasized the critical public health implications of environmental exposure to heavy metals, specifically highlighting the significant associations between the levels of urinary Ba and Cd and HRC, a recognized cardiovascular risk factor. Given the pervasive nature of these metals in the environment, largely attributable to industrial pollution and consumer products, our results call for heightened awareness and preventive strategies. Public health initiatives should prioritize reducing exposure to these metals as part of a broader approach to cardiovascular risk mitigation. Moreover, these findings advocate for the development of clinical guidelines that incorporate environmental exposure assessments in routine cardiovascular risk evaluations. Collaborative efforts between healthcare providers, policymakers, and environmental agencies are essential to address this modifiable risk factor, potentially through stricter regulatory standards and public education on exposure sources.

### 4.6. Strengths and Limitations

There are two notable strengths in this study. First, this is the first research to investigate the relationship between co-exposure to toxic metal mixtures and HRC. Second, we employed four statistical methods to comprehensively evaluate the effects of toxic metal mixtures on the HRC risk across a relatively large population from various perspectives, ensuring the acquisition of reliable results. Nevertheless, this study’s limitations also need to be known. First, due to this study’s cross-sectional design, the sequence of exposure and outcomes was not determined. Second, considering the representativeness of the sample and inclusion and exclusion, potential selective bias may exist in this study. Third, one-time measurement of metal levels in urine does not reflect long-term exposure to metals, and urine as a biomarker can only accurately reflect the level of long-term internal exposure to certain metals, such as Pb, which is more appropriately measured in serum. Finally, some covariables were incomplete and had missing information.

## 5. Conclusions

Our study found a crucial association between toxic metal mixtures and elevated risk of HRC, with Ba and Cd being the primary contributors. Analogous relationships were found in young and middle-aged participants (20 to 59 years), males, and participants with higher BMI status (BMI ≥ 25 kg/m^2^). Our findings indicate that lowering exposure to toxic metals, particularly Ba and Cd, could be crucial in preventing HRC. Future research is warranted to explore the prospective relationship between toxic metal exposure and HRC, as well as to clarify the intricate mechanisms connecting these factors to HRC.

## Figures and Tables

**Table 1:** Basic characteristics of participants by HRC in the U.S. adults (NHANES, 1999–2018).

Characteristics	Total	HRC	Non-HRC	*p* Value
Age, n (%)				<0.001
20–59	3996 (70.23)	937 (77.62)	3059 (80.09)	
≥60	1694 (29.77)	471 (22.38)	1223 (19.91)	
Gender, n (%)				<0.001
Male	2725 (47.89)	785 (58.30)	1940 (43.93)	
Female	2965 (52.11)	623 (41.70)	2342 (56.07)	
Race/ethnicity, n (%)				<0.001
Mexican American	1020 (17.93)	344 (12.31)	676 (8.77)	
Other Hispanic	1217 (21.39)	168 (6.62)	1049 (13.04)	
Non-Hispanic White	2290 (40.25)	604 (67.48)	1686 (65.17)	
Non-Hispanic Black	562 (9.88)	168 (6.56)	394 (5.90)	
Other race/multiracial	601 (10.56)	124 (7.04)	477 (7.12)	
BMI, n (%)				<0.001
Normal or low weight	1754 (30.83)	222 (13.91)	1532 (38.26)	
Overweight	1837 (32.28)	495 (35.85)	1342 (31.06)	
Obese	2034 (35.75)	672 (49.08)	1362 (29.91)	
Missing	65 (1.14)	19 (1.16)	46 (0.76)	
Education level, n (%)				<0.001
Below high school	1506 (26.47)	477 (21.87)	1029 (15.93)	
High school or equivalent	1320 (23.2)	607 (29.05)	962 (22.72)	
Above high school	2864 (50.33)	573 (49.08)	2291 (61.35)	
PIR, n (%)				0.420
0–1.3	1634 (28.72)	435 (21.80)	1199 (19.75)	
1.31–3.50	2055 (36.12)	503 (35.20)	1552 (34.96)	
>3.50	1523 (26.77)	340 (36.57)	1183 (38.52)	
Missing	478 (8.4)	130 (6.44)	348 (6.77)	
Marital status, n (%)				<0.001
Married/cohabiting	3428 (60.25)	893 (65.95)	2535 (63.25)	
Widowed/divorced/separated	1216 (21.37)	335 (20.69)	881 (17.08)	
Never married	1046 (18.38)	180 (13.36)	866 (19.67)	
Serum cotinine, n (%)				0.060
<14 ng/ml	4279 (75.2)	1022 (71.57)	3257 (75.05)	
≥14 ng/ml	1411 (24.8)	386 (28.43)	1025 (24.95)	
Drinking status, n (%)				0.010
Never	730 (12.83)	184 (10.26)	546 (9.68)	
Former	809 (14.22)	247 (14.74)	562 (10.98)	
Now	3555 (62.48)	852 (67.15)	2703 (70.20)	
Missing	596 (10.47)	125 (7.85)	471 (9.13)	
HEI-2015, n (%)				0.870
Inadequate	2703 (47.5)	692 (49.74)	2011 (48.71)	
Average	2183 (38.37)	541 (38.70)	1642 (38.09)	
Optimal	487 (8.56)	118 (7.99)	369 (8.46)	
Missing	317 (5.57)	57 (3.58)	260 (4.74)	
Physical activity, n (%)				0.55
Poor	1219 (21.42)	289 (22.70)	930 (22.28)	
Intermediate	656 (11.53)	149 (11.17)	507 (12.83)	
Ideal	2351 (41.32)	543 (41.88)	1808 (45.19)	
Missing	1464 (27.01)	427 (24.26)	1037 (19.70)	
Hypertension, n (%)				<0.001
No	4989 (87.68)	1138 (85.13)	3851 (93.17)	
Yes	701 (12.32)	270 (14.87)	431 (6.83)	
Diabetes, n (%)				<0.001
No	3544 (62.28)	730 (54.10)	2814 (71.58)	
Yes	2146 (37.72)	678 (45.90)	1468 (28.42)	

Categorical variables were presented as n (%). The chi-square values were calculated without including missing values. Abbreviations: NHANES: National Health and Nutrition Examination Survey; HRC: high remnant cholesterol; BMI: body mass index; PIR: the ratio of family income to poverty; HEI: healthy eating index; n: numbers of subjects; %: weighted percentage.

**Table 2:** Associations of single metals with HRC risk (NHANES, 1999–2018).

Urine Metals (μg/g Creatinine)	Continuous	Q1	Q2	Q3	Q4	*p* for Trend
	OR (95% CI)		OR (95% CI)	OR (95% CI)	OR (95% CI)	
Ba						
Model 1	1.10 (1.03, 1.18)	1.00 (reference)	0.87 (0.67, 1.13)	1.40 (1.11, 1.76)	1.29 (1.01,1.65)	0.003
Model 2	1.11 (1.04, 1.20)	1.00 (reference)	0.94 (0.72, 1.23)	1.49 (1.17, 1.91)	1.33 (1.01, 1.75)	0.005
Cd						
Model 1	1.17 (1.10, 1.25)	1.00 (reference)	1.12 (0.89, 1.40)	1.46 (1.14, 1.87)	1.54 (1.23, 1.93)	<0.001
Model 2	1.16 (1.08, 1.25)	1.00 (reference)	1.08 (0.85, 1.37)	1.38 (1.04, 1.82)	1.50 (1.16, 1.94)	0.002
Co						
Model 1	1.05 (0.94, 1.16)	1.00 (reference)	1.15 (0.96, 1.38)	1.10 (0.85, 1.41)	1.08 (0.84, 1.40)	0.666
Model 2	1.03 (0.93, 1.15)	1.00 (reference)	1.13 (0.92, 1.40)	1.10 (0.82, 1.46)	1.09 (0.82, 1.46)	0.641
Cs						
Model 1	0.98 (0.86, 1.11)	1.00 (reference)	1.18 (0.94, 1.47)	0.94 (0.76, 1.18)	1.00 (0.78, 1.29)	0.668
Model 2	0.94 (0.82, 1.09)	1.00 (reference)	1.12 (0.89, 1.42)	0.86 (0.67, 1.10)	0.97 (0.73, 1.28)	0.477
Hg						
Model 1	1.02 (0.97, 1.09)	1.00 (reference)	1.19 (0.94, 1.49)	1.09 (0.87, 1.36)	1.15 (0.87, 1.36)	0.431
Model 2	1.09 (1.03, 1.16)	1.00 (reference)	1.34 (1.04, 1.71)	1.22 (0.95, 1.57)	1.52 (1.15, 2.01)	0.007
Mo						
Model 1	1.07 (0.98, 1.17)	1.00 (reference)	1.02 (0.79, 1.31)	1.04 (0.83, 1.31)	1.20 (0.98, 1.48)	0.097
Model 2	1.04 (0.94, 1.15)	1.00 (reference)	0.99 (0.76, 1.30)	1.06 (0.82, 1.35)	1.14 (0.90, 1.43)	0.257
Pb						
Model 1	1.08 (1.00, 1.15)	1.00 (reference)	1.19 (0.96, 1.47)	1.33 (1.08, 1.65)	1.28 (1.03, 1.59)	0.014
Model 2	1.09 (1.00, 1.18)	1.00 (reference)	1.20 (0.95, 1.52)	1.37 (1.09, 1.71)	1.35 (1.06, 1.73)	0.007
Sb						
Model 1	0.98 (0.91, 1.05)	1.00 (reference)	1.01 (0.82, 1.26)	1.21 (0.98, 1.50)	0.96 (0.76, 1.20)	0.967
Model 2	1.01 (0.93, 1.09)	1.00 (reference)	1.02 (0.81, 1.27)	1.20 (0.96,1.50)	1.02 (0.81, 1.29)	0.618
Tl						
Model 1	0.99 (0.88, 1.12)	1.00 (reference)	1.17 (0.94, 1.47)	0.95 (0.75, 1.19)	1.04 (0.80, 1.36)	0.883
Model 2	1.05 (0.92, 1.19)	1.00 (reference)	1.25 (0.99, 1.58)	0.97 (0.76, 1.25)	1.18 (0.89,1.56)	0.547
Tu						
Model 1	0.98 (0.92, 1.04)	1.00 (reference)	1.13 (0.90, 1.42)	0.86 (0.69, 1.06)	1.00 (0.80, 1.25)	0.511
Model 2	0.99 (0.92, 1.06)	1.00 (reference)	1.11 (0.87, 1.42)	0.84 (0.66,1.06)	1.01 (0.79, 1.29)	0.602

Abbreviations: Continuous: Ln-transformed concentration of metal; HRC: high remnant cholesterol; Q: quartile; Ba: barium; Cd: cadmium; Co: cobalt; Cs: cesium; Hg: mercury; Mo: molybdenum; Pb: lead; Sb: antimony; Tl: thallium; Tu: tungsten. Model 1 was adjusted for age and gender. Model 2 was adjusted for age, gender, race/ethnicity, body mass index, education level, PIR, marital status, serum cotinine, drinking alcohol status, HEI-2015, physical activity, hypertension, and diabetes.

**Table 3:** Summary results from different models.

Metal	Multivariate Logistic Regression *	WQS (Positive Direction)	WQS (Negative Direction)	qgcomp	BKMR (PIP)
Ba	+	0.192	0.000	0.307 (+)	1.000
Cd	+	0.278	0.004	0.215 (+)	0.869
Co		0.003	0.291	0.302 (−)	0.093
Cs		0.000	0.484	0.570 (−)	0.552
Hg	+	0.253	0.001	0.168 (+)	0.235
Mo		0.040	0.058	0.053 (+)	0.792
Pb	+	0.077	0.012	0.189 (+)	0.119
Sb		0.078	0.026	0.128 (−)	0.082
Tl		0.019	0.081	0.047 (+)	0.084
Tu		0.061	0.043	0.022 (+)	0.010

Abbreviations: WQS: weighted quantile sum; qgcomp: quantile g-computation; BKMR: Bayesian kernel machine regression. PIP: posterior inclusion probability. “*” means that compared with participants in the lowest quartile of measured metal, participants in the highest quartile of measured metal were significantly associated with HRC risk with adjustment for covariates (model 2). “+” means positive weight, while “−” means negative weight.

## Data Availability

The data presented in this study are publicly accessible on the NHANES website [https://www.cdc.gov/nchs/nhanes/index.htm] (accessed on 31 May 2023).

## Supplementary Materials

The following supporting information can be downloaded at https://www.mdpi.com/article/10.3390/toxics12060430/s1. Figure S1: Flow chart population included in this analysis (N = 5690), NHANES, USA; Figure S2: Pearson’s correlation matrix among Ln-transformed urinary metals in the study population; Figure S3: univariate exposure–response functions and the bivariate exposure–response function using Bayesian kernel machine regression; Figure S4: Odds ratios (95 % CI) of HRC associated with co-exposure to urinary metal mixtures by WQS (A) and qgcomp (B) analyses in different subgroups; Figure S5: Combined effects of metals mixture on HRC risk in different subgroups were estimated by Bayesian Kernel Machine Regression (BKMR) models; Figure S6: Estimated risk and weights of each metal in positive and negative WQS and qgcomp models after multiple imputation of covariates with missing values; Figure S7: Effects of urinary metals mixture on HRC risk in total population were estimated by Bayesian Kernel Machine Regression (BKMR) models after multiple imputation of covariates with missing values. Table S1: Distributions of urine metals in the study population; Table S2: Associations of single metals with HRC risk in subjects with co-exposure of urine metals; Table S3: Associations of single metals with HRC risk using false discovery rate to adjust P-values; Table S4: Associations of single urinary metals with HRC risk after multiple imputation of covariates with missing values.
