# Supplementary material for: Associations of Urinary Heavy Metal Mixtures with High Remnant Cholesterol among US Adults: Evidence from the National Health and Nutrition Examination Survey (1998–2018)
Source: Toxics. 2024 Jun 13;12(6):430. doi: 10.3390/toxics12060430 (PMC11209470; doi:10.3390/toxics12060430)
Supplement: Supplementary file 1 [file toxics-12-00430-s001.zip › supplementary data.pdf]

Supplementary data

Associations of Urinary Heavy Metal Mixtures with High Remnant Cholesterol among US Adults: Evidence from the National Health and Nutrition Examination Survey (1998–2018)

Hui Li †, Bei-Jing Cheng †, Pei-Yan Yang, Chun Wang, Ke Meng, Tian-Lin Li, Jia Wang and Ran Liu *

Key Laboratory of Environmental Medicine Engineering, Ministry of Education, School of Public Health, Southeast University, Nanjing 210009, China

* Correspondence: ranliu@seu.edu.cn; Tel.: +86-13813373523

† These authors contributed equally to this work.

Supplementary tables

Table S1 Distributions of urine metals in the study population.

Table S2 Associations of single metals with HRC risk in subjects with co-exposure of urine metals.

Table S3 Associations of single metals with HRC risk using false discovery rate to adjust P-values.

Table S4 Associations of single metals with HRC risk after multiple imputation of covariates with missing values.

Table S1. Distributions of urine metals in the study population.

Urine (μg/L)	Detection rate (%)	Median	Interquartile range
Ba	98.55	1.100	0.560-2.087
Cd	97.08	0.290	0.147-0.540
Co	99.21	0.349	0.214-0.540
Cs	99.96	4.610	2.852-6.890
Hg	84.62	0.390	0.170-0.900
Mo	99.95	40.400	22.400-66.380
Pb	96.96	0.470	0.250-0.820
Sb	74.01	0.052	0.029-0.094
Tl	99.65	0.169	0.102-0.260
Tu	84.09	0.063	0.030-0.120

Abbreviations: Ba: Barium; Cd: cadmium; Co: cobalt; Cs: cesium; Hg: mercury; Mo: molybdenum; Pb: lead; Sb: antimony; Tl: thallium; Tu: tungsten.

Table S2. Associations of single metals with HRC risk in subjects with co-exposure of urine metals.

Urine metals (μg/g creatinine)	Continuous OR (95% CI)	Q1	Q2 OR (95% CI)	Q3 OR (95% CI)	Q4 OR (95% CI)	*p* for trend
Ba	**1.13 (1.05, 1.21)**	1.00 (reference)	0.92 (0.71, 1.20)	**1.47 (1.15, 1.88)**	**1.32 (1.00, 1.75)**	**0.009**
Cd	**1.16 (1.06, 1.26)**	1.00 (reference)	1.01 (0.78, 1.31)	1.30 (0.96, 1.77)	**1.44 (1.07, 1.95)**	**0.010**
Co	0.98 (0.87, 1.10)	1.00 (reference)	1.03 (0.83, 1.29)	0.99 (0.74, 1.32)	0.94 (0.69, 1.27)	0.604
Cs	**0.80 (0.67, 0.94)**	1.00 (reference)	0.99 (0.76, 1.29)	**0.70 (0.54, 0.92)**	**0.71 (0.51, 0.99)**	**0.014**
Hg	**1.08 (1.01, 1.16)**	1.00 (reference)	**1.32 (1.03, 1.70)**	1.20 (0.91, 1.59)	**1.44 (1.06, 1.94)**	**0.037**
Mo	1.03 (0.92, 1.15)	1.00 (reference)	0.98 (0.75, 1.29)	1.03 (0.80, 1.34)	1.13 (0.89, 1.44)	0.301
Pb	1.02 (0.92, 1.12)	1.00 (reference)	1.14 (0.88, 1.48)	1.24 (0.95, 1.62)	1.16 (0.85, 1.57)	0.295
Sb	0.97 (0.94, 1.06)	1.00 (reference)	0.98 (0.78, 1.23)	1.12 (0.90, 1.40)	0.93 (0.72, 1.19)	0.702
TI	1.09 (0.94, 1.25)	1.00 (reference)	**1.28 (1.01, 1.64)**	1.03 (0.78, 1.36)	1.26 (0.92, 1.73)	0.342
Tu	0.96 (0.89, 1.03)	1.00 (reference)	1.10 (0.85, 1.41)	**0.77 (0.60, 0.99)**	0.91 (0.70, 1.18)	0.183

Abbreviations: Continuous: Ln-transformed concentration of metal; HRC: high remnant cholesterol; Q: quartile; Ba: Barium; Cd: cadmium; Co: cobalt; Cs: cesium; Hg: mercury; Mo: molybdenum; Pb: lead; Sb: antimony; Tl: thallium; Tu: tungsten.

Models 2 were adjusted for age, gender, race/ethnicity, body mass index, education level, PIR, marital status, serum cotinine, drinking status, HEI-2015, Physical activity, Hypertension and Diabetes.

Table S3 Associations of single metals with HRC risk using false discovery rate to adjust P-values.

Variables	Model 1 [a]			Model 2 [b]		
	OR (95%CI)	*P*-value	*P*-value [c]	OR (95%CI)	*P*-value	*P*-value [c]
Ba						
Q1	1.00	–	–	1.00	–	–
Q2	0.87 (0.67,1.13)	0.302	0.906	0.94 (0.72,1.23)	0.664	1.000
Q3	**1.40 (1.11,1.76)**	**0.005**	**0.015**	1.49 (1.17,1.91)	**0.002**	**0.006**
Q4	**1.29 (1.01,1.65)**	**0.044**	0.132	1.33 (1.01,1.75)	**0.043**	0.129
P-trend	**0.003**			**0.005**		
Cd						
Q1	1.00	–	–	1.00	–	–
Q2	1.12 (0.89,1.40)	0.350	1.000	1.08 (0.85,1.37)	0.546	1.000
Q3	**1.46 (1.14,1.87)**	**0.004**	**0.012**	**1.38 (1.04,1.82)**	**0.028**	0.084
Q4	**1.54 (1.23,1.93)**	**<0.001**	**0.003**	**1.50 (1.16,1.94)**	**0.003**	**0.009**
P-trend	**<0.001**			**0.002**		
Co						
Q1	1.00	–	–	1.00	–	–
Q2	1.15 (0.96,1.38)	0.133	0.399	1.13 (0.92,1.40)	0.244	0.732
Q3	1.10 (0.85,1.41)	0.474	1.000	1.10 (0.82,1.46)	0.522	1.000
Q4	1.08 (0.84,1.40)	0.535	1.000	1.09 (0.82,1.46)	0.539	1.000
P-trend	0.666			0.641		
Cs						
Q1	1.00	–	–	1.00	–	–
Q2	1.18 (0.94,1.47)	0.150	0.450	1.12 (0.89,1.42)	0.330	0.990
Q3	0.94 (0.76,1.18)	0.608	1.000	0.86 (0.67,1.10)	0.239	0.717
Q4	1.00 (0.78,1.29)	0.973	1.000	0.97 (0.73,1.28)	0.815	1.000
P-trend	0.668			0.477		
Hg						
Q1	1.00	–	–	1.00	–	–
Q2	1.19 (0.94,1.49)	0.142	0.426	**1.34 (1.04,1.71)**	**0.022**	0.066
Q3	1.09 (0.87,1.36)	0.468	1.000	1.22 (0.95,1.57)	0.125	0.375
Q4	1.15 (0.87,1.36)	0.306	0.918	**1.52 (1.15,2.01)**	**0.003**	**0.009**
P-trend	0.431			**0.007**		
Mo						

Q1	1.00	–	–	1.00	–	–
Q2	1.02 (0.79,1.31)	0.875	1.000	0.99 (0.76,1.30)	0.964	1.000
Q3	1.04 (0.83,1.31)	0.704	1.000	1.06 (0.82,1.35)	0.662	1.000
Q4	1.20 (0.98,1.48)	0.079	0.237	1.14 (0.90,1.43)	0.276	0.828
P-trend	0.097			0.257		
Pb						
Q1	1.00	–	–	1.00	–	–
Q2	1.19 (0.96,1.47)	0.106	0.318	1.20 (0.95,1.52)	0.127	0.381
Q3	**1.33 (1.08,1.65)**	**0.009**	**0.027**	**1.37 (1.09,1.71)**	**0.007**	**0.021**
Q4	**1.28 (1.03,1.59)**	**0.026**	0.078	**1.35 (1.06,1.73)**	**0.015**	**0.045**
P-trend	**0.014**			**0.007**		
Sb						
Q1	1.00	–	–	1.00	–	–
Q2	1.01 (0.82,1.26)	0.896	1.000	1.02 (0.81,1.27)	0.889	1.000
Q3	1.21 (0.98,1.50)	0.078	0.234	1.20 (0.96,1.50)	0.112	0.336
Q4	0.96 (0.76,1.20)	0.694	1.000	1.02 (0.81,1.29)	0.853	1.000
P-trend	0.967			0.618		
Tl						
Q1	1.00	–	–	1.00	–	–
Q2	1.17 (0.94,1.47)	0.161	0.483	1.25 (0.99,1.58)	0.060	0.180
Q3	0.95 (0.75,1.19)	0.634	1.000	0.97 (0.76,1.25)	0.838	1.000
Q4	1.04 (0.80,1.36)	0.758	1.000	1.18 (0.89,1.56)	0.257	0.771
P-trend	0.883			0.547		
Tu						
Q1	1.00	–	–	1.00	–	–
Q2	1.13 (0.90,1.42)	0.295	0.885	1.11 (0.87,1.42)	0.390	1.000
Q3	0.86 (0.69,1.06)	0.152	0.456+	0.84 (0.66,1.06)	0.135	0.405
Q4	1.00 (0.80,1.25)	0.991	1.000	1.01 (0.79,1.29)	0.937	1.000
P-trend	0.511			0.602		

Abbreviations: Continuous: Ln-transformed concentration of metal; HRC: high remnant cholesterol; Q: quartile; Ba: Barium; Cd: cadmium; Co: cobalt; Cs: cesium; Hg: mercury; Mo: molybdenum; Pb: lead; Sb: antimony; Tl: thallium; Tu: tungsten.

[a] Adjusting age and gender.

[b] Adjusting age, gender, race/ethnicity, body mass index, education level, PIR, marital status, serum cotinine, drinking status, HEI-2015, Physical activity, Hypertension, and Diabetes.

[c] False Discovery Rate (Bonferroni).

Table S4. Associations of single urinary metals with HRC risk after multiple imputation of covariates with missing values.

Metals	Continuous<br>OR (95% CI)	Q1	Q2<br>OR (95% CI)	Q3<br>OR (95% CI)	Q4<br>OR (95% CI)	*p* for trend
Ba						
Model 1	**1.10 (1.03,1.18)**	1.00 (reference)	0.87 (0.67,1.13)	**1.40 (1.11,1.76)**	**1.29 (1.01,1.65)**	**0.003**
Model 2	**1.12 (1.04,1.20)**	1.00 (reference)	0.95 (0.73,1.23)	**1.51 (1.18,1.93)**	**1.34 (1.01,1.76)**	**0.005**
Cd						
Model 1	**1.17 (1.10,1.25)**	1.00 (reference)	1.11 (0.89,1.39)	**1.45 (1.13,1.86)**	**1.53 (1.22,1.92)**	**0.001**
Model 2	**1.16 (1.08,1.25)**	1.00 (reference)	1.08 (0.85,1.36)	**1.38 (1.05,1.83)**	**1.49 (1.15,1.93)**	**0.001**
Co						
Model 1	1.05 (0.94,1.16)	1.00 (reference)	1.15 (0.96,1.38)	1.10 (0.85,1.41)	1.08 (0.84,1.40)	0.666
Model 2	1.03 (0.93,1.15)	1.00 (reference)	1.13 (0.92,1.40)	1.10 (0.83,1.47)	1.10 (0.83,1.46)	0.609
Cs						
Model 1	0.98 (0.86,1.11)	1.00 (reference)	1.18 (0.94,1.47)	0.83 (0.61, 1.12)	1.03 (0.74, 1.42)	0.608
Model 2	0.94 (0.81,1.09)	1.00 (reference)	1.11 (0.88,1.41)	0.86 (0.67,1.10)	0.96 (0.73,1.28)	0.467
Hg						
Model 1	1.02 (0.97,1.09)	1.00 (reference)	1.19 (0.94,1.49)	0.94 (0.76,1.18)	1.00 (0.78,1.29)	0.431
Model 2	**1.09 (1.02,1.16)**	1.00 (reference)	**1.33 (1.04,1.70)**	1.21 (0.94,1.55)	**1.51 (1.14,1.99)**	**0.009**
Mo						
Model 1	1.07 (0.98,1.17)	1.00 (reference)	1.02 (0.79,1.31)	1.04 (0.83,1.31)	1.20 (0.98,1.48)	0.093
Model 2	1.05 (0.95,1.16)	1.00 (reference)	1.00 (0.77,1.30)	1.06 (0.83,1.36)	1.14 (0.91,1.44)	0.240
Pb						
Model 1	**1.08 (1.00,1.15)**	1.00 (reference)	1.19 (0.96,1.47)	**1.33 (1.08,1.65)**	**1.28 (1.03,1.59)**	**0.014**
Model 2	**1.09 (1.00,1.18)**	1.00 (reference)	1.20 (0.95,1.51)	**1.38 (1.10,1.72)**	**1.36 (1.07,1.73)**	**0.006**
Sb						
Model 1	0.98 (0.91,1.05)	1.00 (reference)	1.01 (0.82,1.26)	1.21 (0.98,1.50)	0.96 (0.76,1.20)	0.880
Model 2	1.00 (0.93,1.09)	1.00 (reference)	1.01 (0.81,1.27)	1.19 (0.95,1.49)	1.02 (0.81,1.28)	0.662
Tl						
Model 1	0.99 (0.88,1.12)	1.00 (reference)	1.17 (0.94,1.47)	0.95 (0.75,1.19)	1.04 (0.80,1.36)	0.804
Model 2	1.05 (0.92,1.19)	1.00 (reference)	1.25 (0.99,1.58)	0.97 (0.76,1.25)	1.17 (0.88,1.55)	0.596
Tu						
Model 1	0.98 (0.92,1.04)	1.00 (reference)	1.13 (0.90,1.42)	0.86 (0.69,1.06)	1.00 (0.80,1.25)	0.414
Model 2	0.99 (0.92,1.06)	1.00 (reference)	1.11 (0.87,1.42)	0.84 (0.66,1.06)	1.02 (0.80,1.29)	0.631

Continuous, Ln-transformed concentration of metal; HRC, high remnant cholesterol; Q, quartile.

Model 1 were adjusted for age and gender.

Models 2 were adjusted for age, gender, race/ethnicity, body mass index, education level, PIR, marital status, serum cotinine, drinking status, HEI-2015, Physical activity, Hypertension and Diabetes.

Supplementary figures

Figure S1. Flow chart population included in this analysis (N=5690), NHANES, USA

Figure S2. Pearson's correlation matrix among Ln-transformed urinary metals in the study population.

Figure S3. Univariate exposure–response functions and the bivariate exposure–response function using Bayesian kernel machine regression (BKMR). **Figure S3A** shows univariate exposure–response functions and 95% confidence intervals for associations between single metal and the risk of HRC examined using BKMR when other metals were fixed at the median. **Figure S3B** shows interaction effect of two heavy metals examined using the bivariate exposure–response function of BKMR when the second metal was fixed at its 25th, 50th, and 75th percentiles, respectively.

Figure S4. Odds ratios (95 % CI) of HRC associated with co-exposure to urinary metal mixtures by WQS (**A**) and qgcomp (**B**) analyses in different subgroups. Models were adjusted variables included age, gender, race/ethnicity, body mass index, education level, PIR, marital status, serum cotinine, drinking status, HEI-2015, Physical activity, Hypertension and Diabetes.

Figure S5. Combined effects of metals mixture on HRC risk in different subgroups were estimated by Bayesian Kernel Machine Regression (BKMR) models. **Fig. S5A** shows the age subgroups divided into 60 years (**A1**: 20-59 years, **A2** ≥ 60 years). **Fig. S5B** shows the gender subgroups (**B1**: Male, **B2**: Female). **Fig. 5C** shows the BMI subgroups divided into 25 kg/m^2 (C1: < 25 kg/m^2, C1 ≥ 25 kg/m^2). Models were adjusted variables included age, gender, race/ethnicity, body mass index, education level, PIR, marital status, serum cotinine, drinking status, HEI-2015, Physical activity, Hypertension and Diabetes.

Figure S6. Estimated risk and weights of each metal in positive and negative WQS and qgcomp models after multiple imputation of covariates with missing values. **Figure S6A** shows Odds

ratios (95 % CI) of HRC associated with co-exposure to urinary metal mixtures by WQS and qgcomp analyses after multiple imputation of covariates with missing values. Weighted values of urinary metals for HRC in WQS (**B**) and qgcomp (**C**) models. Models were adjusted variables included age, gender, race/ethnicity, body mass index, education level, PIR, marital status, serum cotinine, drinking status, HEI-2015, Physical activity, Hypertension and Diabetes.

Figure S7. Effects of urinary metals mixture on HRC risk in total population were estimated by Bayesian Kernel Machine Regression (BKMR) models after multiple imputation of covariates with missing values. Univariate exposure-response functions and 95% confidence interval for each metal with the other metals fixed at the median (**A**). Combined effects of the metals as a mixture on HRC risk (**B**). Single-exposure effects and 95% confidence interval with the other metals fixed at a specific quantile (the 25th, 50th, or 75th percentile) (**C**). Bivariate exposure response functions for interaction between any two metals (**D**). Models were adjusted variables included age, gender, race/ethnicity, body mass index, education level, PIR, marital status, serum cotinine, drinking status, HEI-2015, Physical activity, Hypertension and Diabetes.

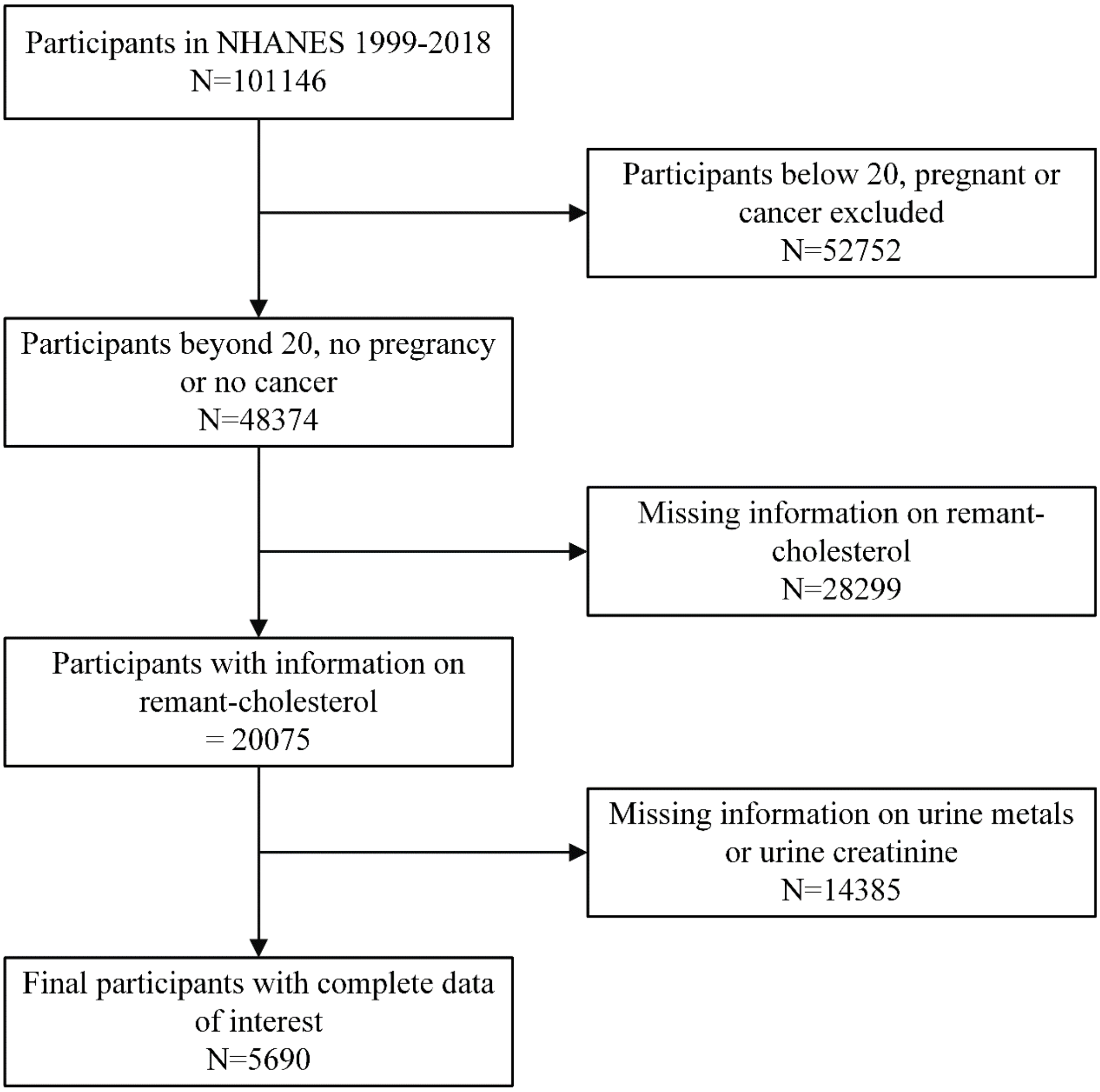

Figure S1. Flow chart population included in this analysis (N=5690), NHANES, USA

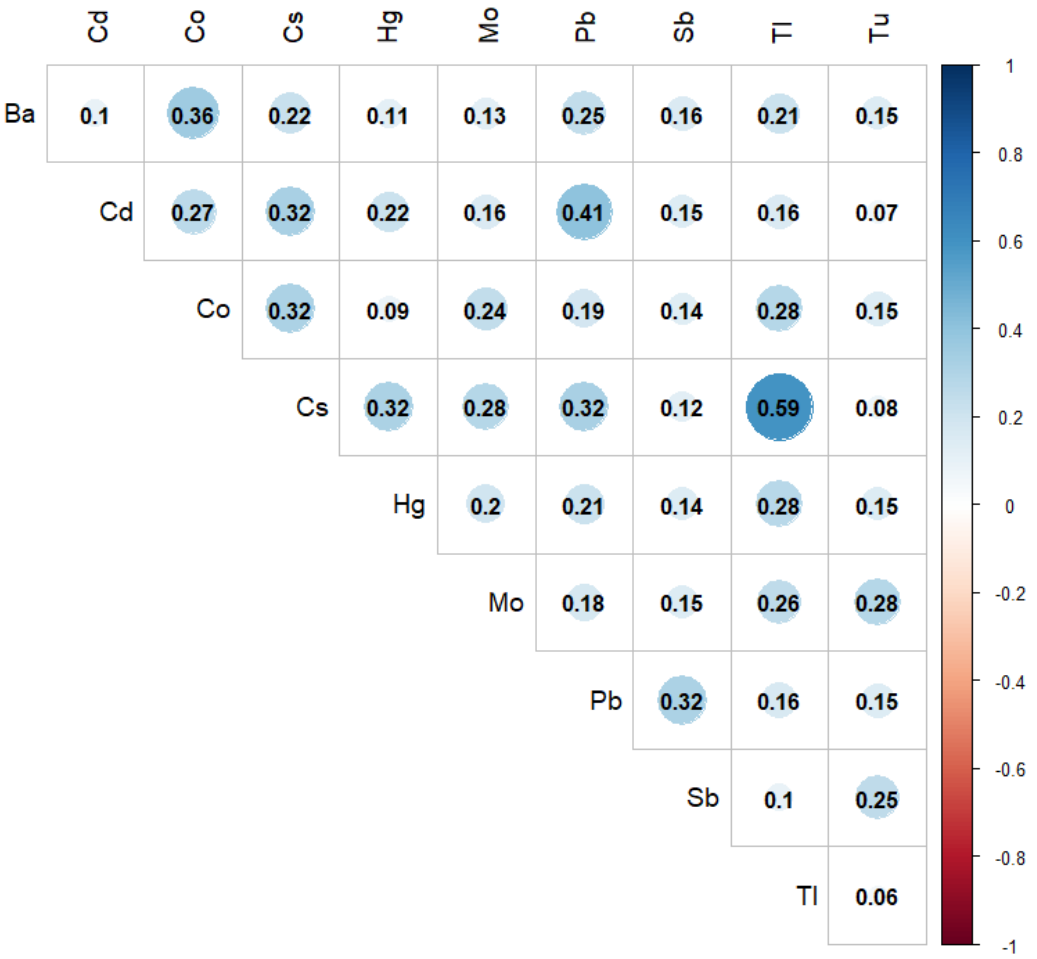

Figure S2. Pearson's correlation matrix among Ln-transformed urinary metals in the study population.

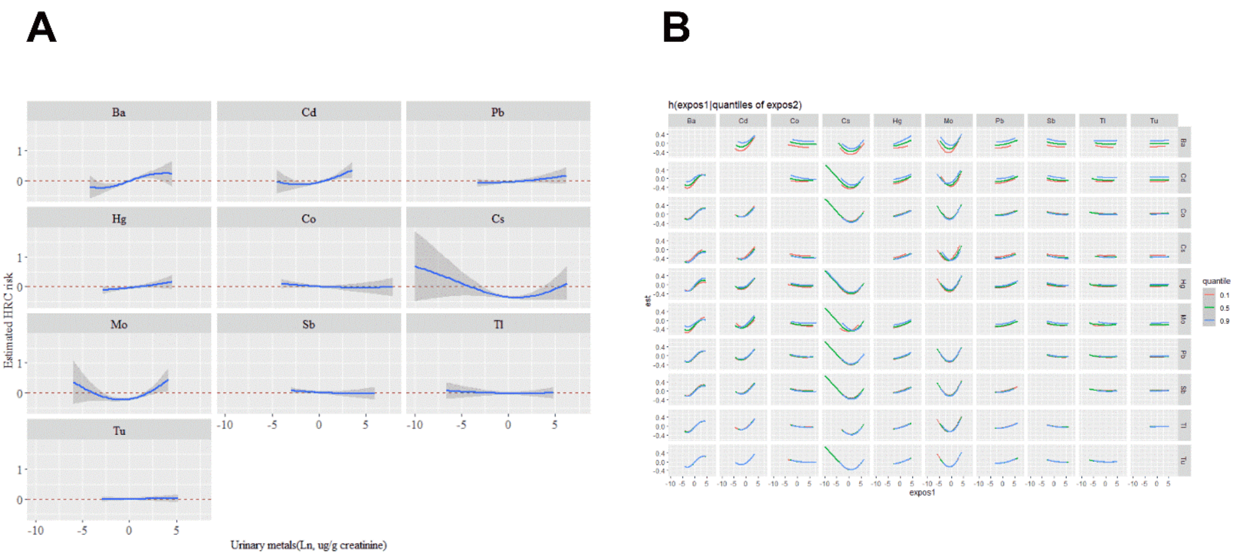

Figure S3. Univariate exposure–response functions and the bivariate exposure–response function using Bayesian kernel machine regression (BKMR). **Figure S3A** shows univariate exposure–response functions and 95% confidence intervals for associations between single metal and the risk of HRC examined using BKMR when other metals were fixed at the median. **Figure S3B** shows interaction effect of two heavy metals examined using the bivariate exposure–response function of BKMR when the second metal was fixed at its 25th, 50th, and 75th percentiles, respectively.

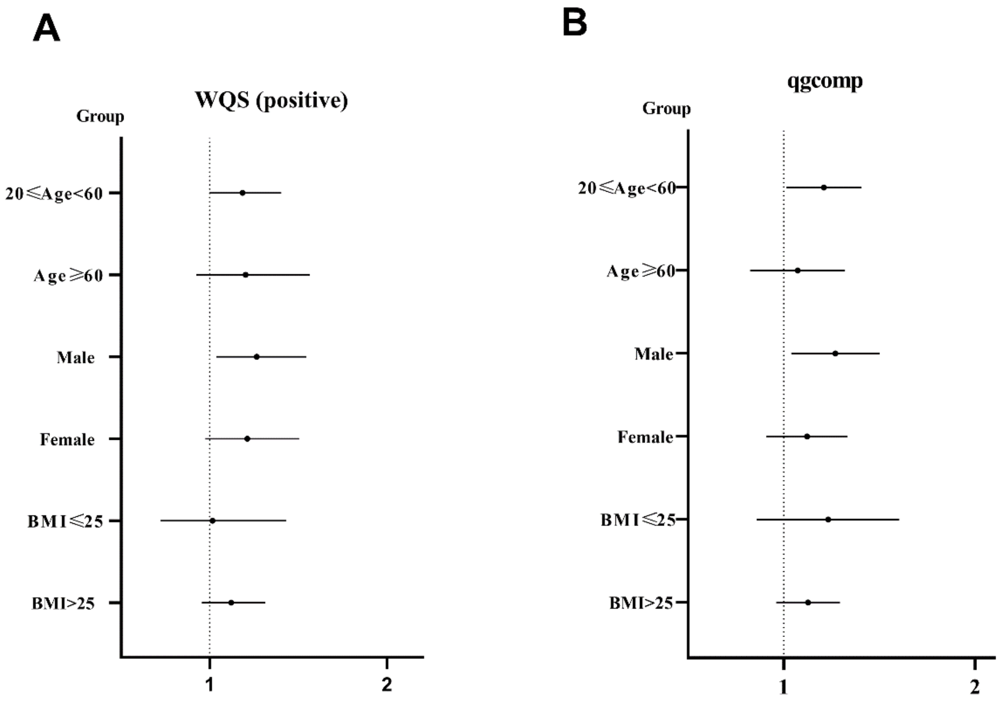

Figure S4. Odds ratios (95 % CI) of HRC associated with co-exposure to urinary metal mixtures by WQS (**A**) and qgcomp (**B**) analyses in different subgroups. Models were adjusted variables included age, gender, race/ethnicity, body mass index, education level, PIR, marital status, serum cotinine, drinking status, HEI-2015, Physical activity, Hypertension and Diabetes.

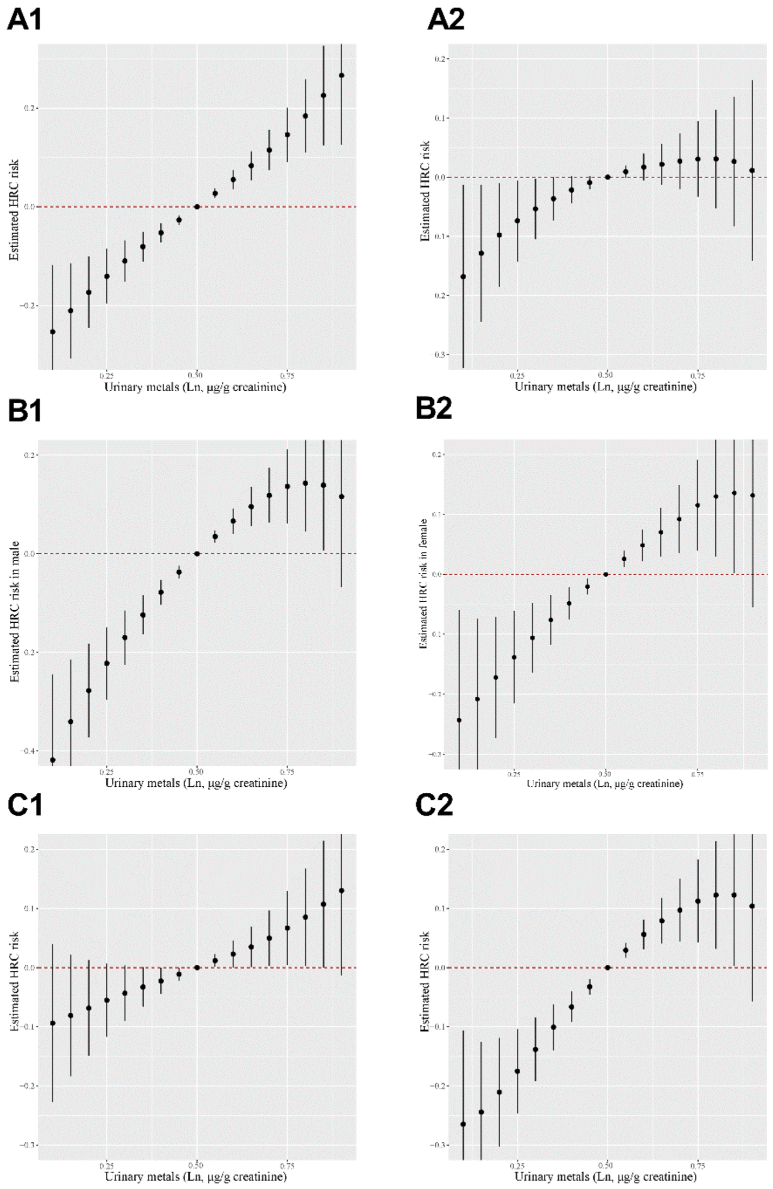

Figure S5. Combined effects of metals mixture on HRC risk in different subgroups were estimated by Bayesian Kernel Machine Regression (BKMR) models. **Fig. S5A** shows the age subgroups divided into 60 years (**A1**: 20-59 years, **A2** ≥ 60 years). **Fig. S5B** shows the gender subgroups (**B1**: Male, **B2**: Female). **Fig. 5C** shows the BMI subgroups divided into 25 kg/m^2 (C1: < 25 kg/m^2, C1 ≥ 25 kg/m^2). Models were adjusted variables included age, gender, race/ethnicity, body mass index, education level, PIR, marital status, serum cotinine, drinking status, HEI-2015, Physical activity, Hypertension and Diabetes.

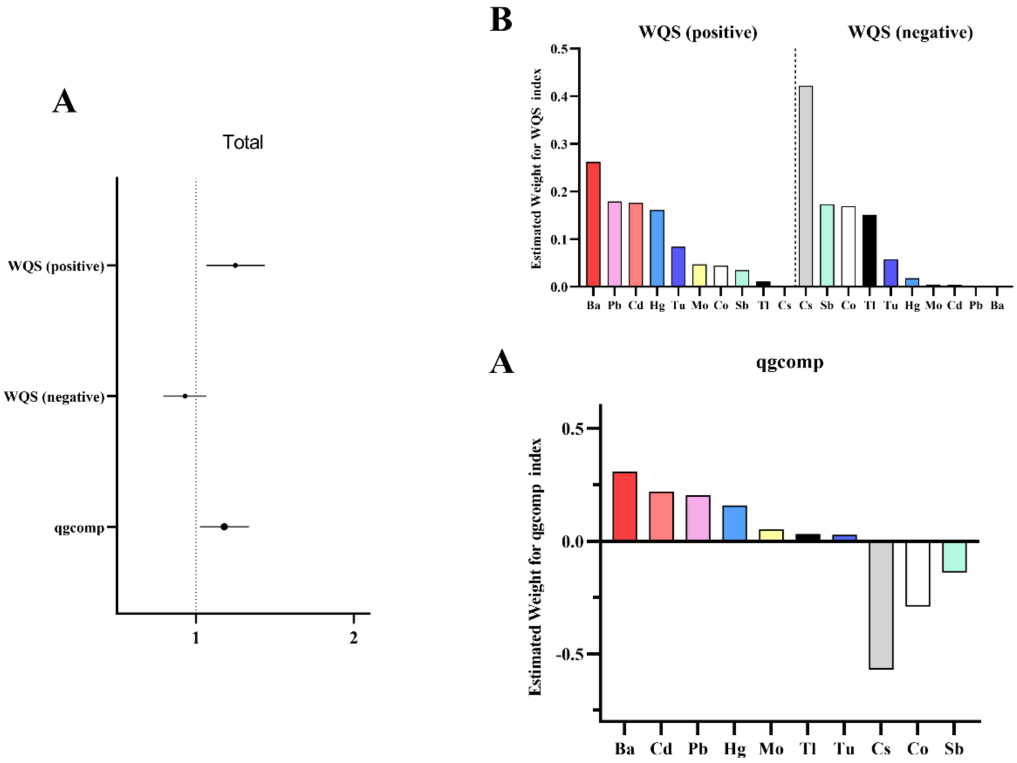

Figure S6. Estimated risk and weights of each metal in positive and negative WQS and qgcomp models after multiple imputation of covariates with missing values. **Figure S6A** shows Odds ratios (95 % CI) of HRC associated with co-exposure to urinary metal mixtures by WQS and qgcomp analyses after multiple imputation of covariates with missing values. Weighted values of urinary metals for HRC in WQS (**B**) and qgcomp (**C**) models. Models were adjusted variables included age, gender, race/ethnicity, body mass index, education level, PIR, marital status, serum cotinine, drinking status, HEI-2015, Physical activity, Hypertension and Diabetes.

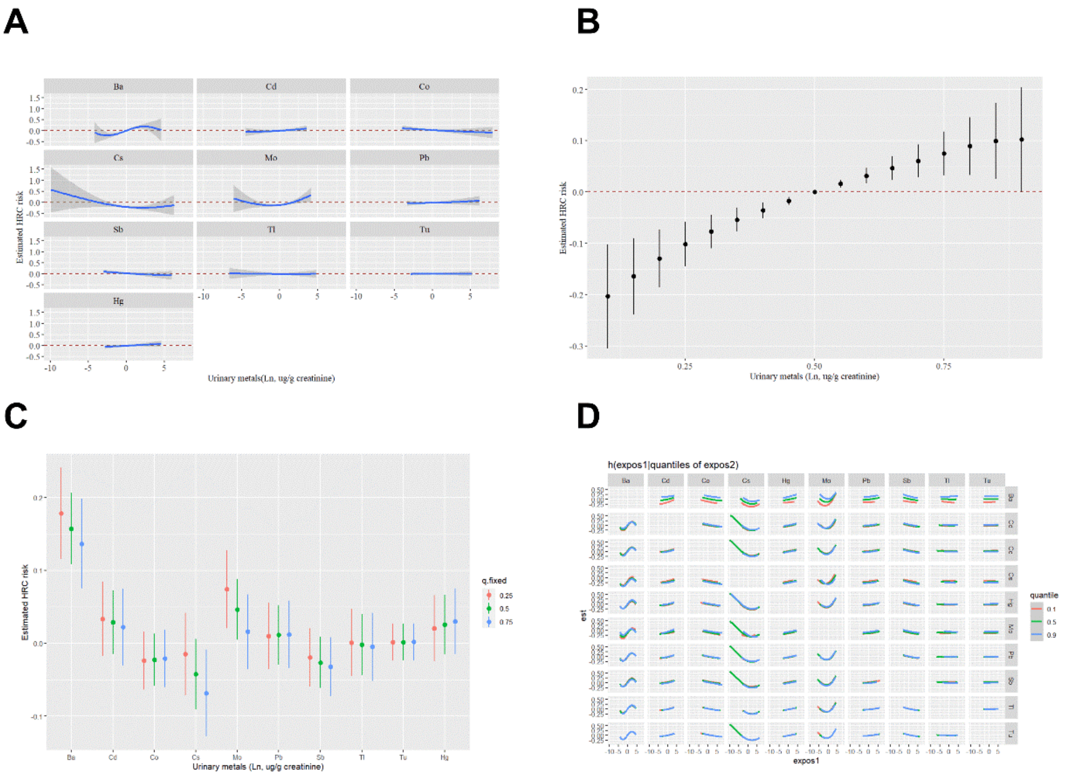

Figure S7. Effects of urinary metals mixture on HRC risk in total population were estimated by Bayesian Kernel Machine Regression (BKMR) models after multiple imputation of covariates with missing values. Univariate exposure-response functions and 95% confidence interval for each metal with the other metals fixed at the median (**A**). Combined effects of the metals as a mixture on HRC risk (**B**). Single-exposure effects and 95% confidence interval with the other metals fixed at a specific quantile (the 25th, 50th, or 75th percentile) (**C**). Bivariate exposure response functions for interaction between any two metals (**D**). Models were adjusted variables included age, gender, race/ethnicity, body mass index, education level, PIR, marital status, serum cotinine, drinking status, HEI-2015, Physical activity, Hypertension and Diabetes.
