# Supplementary figures and images for: Associations of Urinary Heavy Metal Mixtures with High Remnant Cholesterol among US Adults: Evidence from the National Health and Nutrition Examination Survey (1998–2018)
Source: Toxics. 2024 Jun 13;12(6):430. doi: 10.3390/toxics12060430 (PMC11209470; doi:10.3390/toxics12060430)

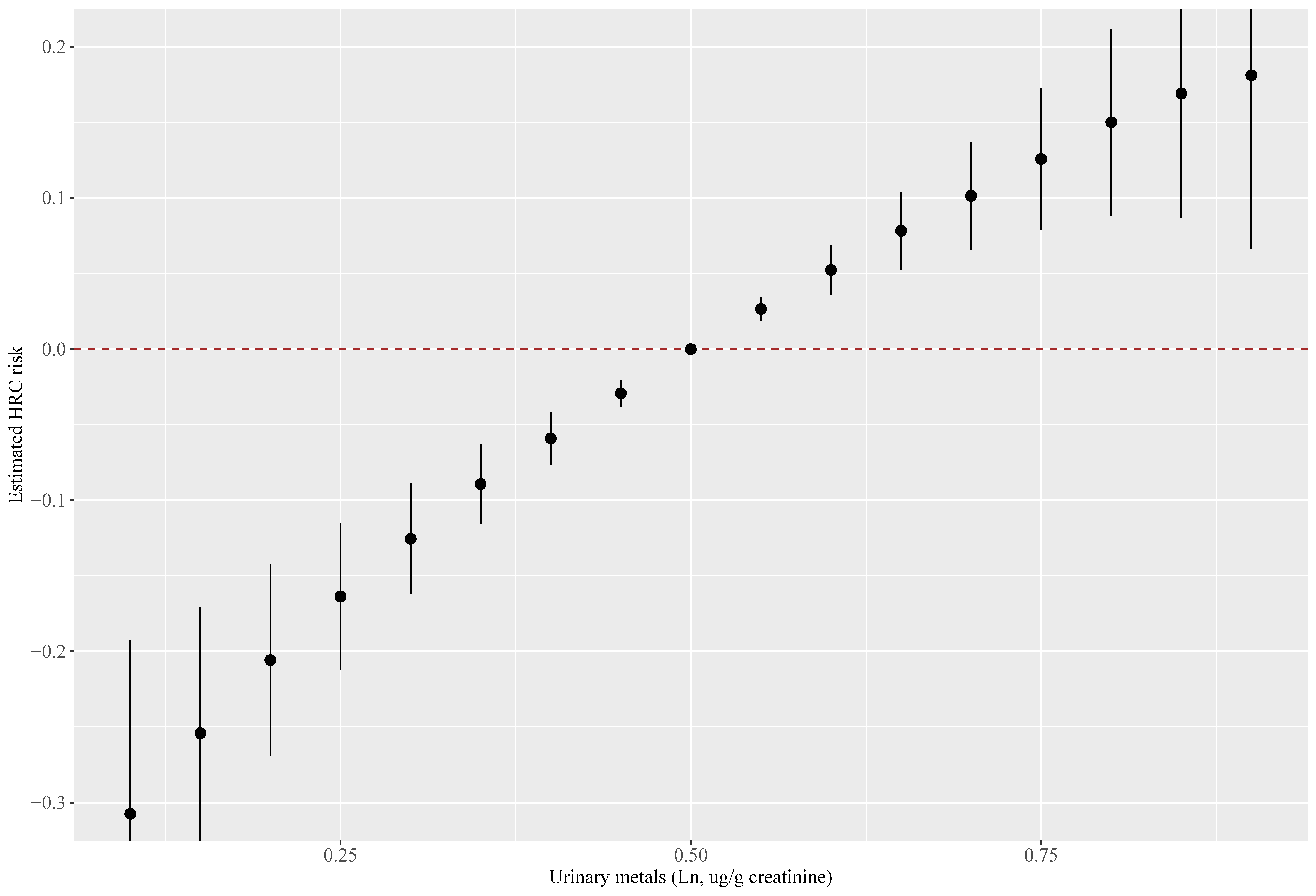

Supplement: Supplementary file 1 [file toxics-12-00430-s001.zip › A.tiff]

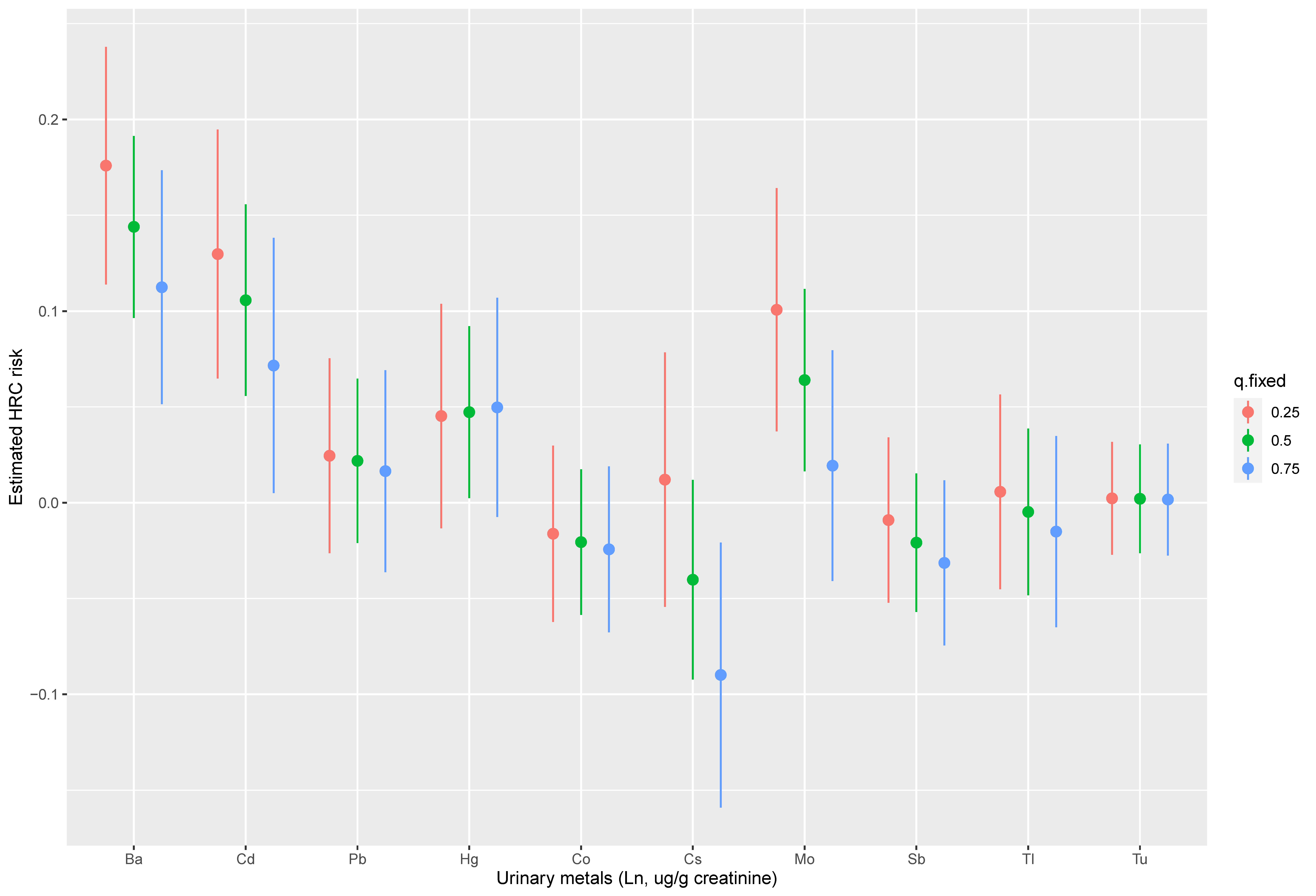

Supplement: Supplementary file 1 [file toxics-12-00430-s001.zip › B.tiff]
